# Loss of NcBPK1 impairs bradyzoite differentiation and enhances virulence in *Neospora caninum*

**DOI:** 10.1186/s13071-025-07076-4

**Published:** 2025-10-21

**Authors:** Rafael Amieva, Laura Rico-San Román, Iván Pastor-Fernández, Andrew Hemphill, Ghalia Boubaker, Esther Collantes-Fernández, Luis Miguel Ortega-Mora, Pilar Horcajo

**Affiliations:** 1 SALUVET Group, Animal Health Department, Faculty of Veterinary Sciences, Complutense University of Madrid, Madrid, Spain; 2 Parasitology Unit, Microbiology and Parasitology Department, Faculty of Pharmacy, Complutense University of Madrid, Madrid, Spain; 3 Institute for Parasitology, Vetsuisse Faculty, University of Bern, Bern, Switzerland

**Keywords:** *Neospora caninum*, BPK1, CRISPR/Cas9, BALB/c, Bovine macrophages, Bradyzoite differentiation

## Abstract

**Background:**

*Neospora caninum* is an apicomplexan parasite responsible for bovine neosporosis, a disease that leads to substantial economic losses in cattle due to abortion and reduced productivity. The pathogenesis of *N*. *caninum* is shaped by complex host–parasite interactions, and virulence is known to vary between strains. BPK1 (Bradyzoite pseudokinase 1), a pseudokinase previously identified as a potential virulence factor in *Toxoplasma gondii*, has not yet been functionally characterized in *N*. *caninum*.

**Methods:**

To investigate the role of NcBPK1 in parasite virulence, a knockout strain (*Nc*Δ*BPK1*) was generated using CRISPR/Cas9 genome editing. The virulence of the mutant was evaluated in a pregnant mouse model by assessing neonatal survival and parasite burden in dam tissues. In vitro assays were conducted to examine parasite replication in bovine macrophages and to analyze the expression of stage-specific genes.

**Results:**

Deletion of *NcBpk1* resulted in enhanced parasite virulence in vivo, as shown by a decrease in neonatal survival and higher parasite loads in maternal brain tissue. The *Nc*Δ*BPK1* mutant also displayed enhanced replication in bovine macrophages and reduced expression of bradyzoite-specific genes, suggesting a defect in stage conversion.

**Conclusions:**

These findings indicate that NcBPK1 is crucial for regulating the balance between acute replication and chronic persistence. Its absence promotes rapid tachyzoite proliferation and worsens disease outcomes. This study sheds light on the molecular mechanisms underlying *N*. *caninum* virulence. Further research is needed to elucidate the signaling pathways and protein interactions involving NcBPK1.

**Graphical Abstract:**

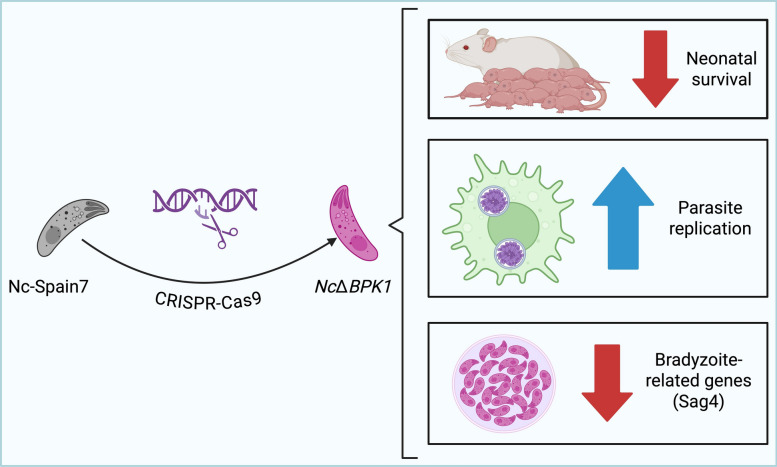

**Supplementary Information:**

The online version contains supplementary material available at 10.1186/s13071-025-07076-4.

## Background

*Neospora caninum* is an obligate intracellular apicomplexan parasite and the causative agent of bovine neosporosis, a leading infectious cause of abortion in cattle worldwide [1]. Economic losses associated with *N*. *caninum *infections arise from reproductive failure, decreased milk production, and increased culling rates [2]. Unlike its close relative *Toxoplasma gondii*, which can infect a broad range of hosts [3], *N*. *caninum* primarily affects canids and ruminants [4].

The pathogenesis of disease caused by apicomplexan parasites is fundamentally dependent on the complex host–parasite interactions [5, 6]. These parasites employ specialized organelles, such as rhoptries and dense granules, to secrete effector proteins that modulate key processes, including cell invasion, immune evasion and intracellular survival [7–12]. On the other hand, virulence in *N*. *caninum* is strain-dependent, with significant intra-specific variation observed despite the relatively low genetic diversity of this parasite [13]. Highly virulent isolates exhibit increased invasion rates and replication efficiency in vitro, traits that correlate with their virulence in vivo [14]. Furthermore, these differences in virulence are reflected in the parasite interactions with innate immune cells, such as bovine macrophages, which play a crucial role in early infection control [15]. However, the full repertoire of virulence-associated proteins and their precise contributions to parasite fitness and pathogenesis remain largely unknown.

Recent comparative transcriptomic and proteomic studies have provided insights into the molecular basis of *N*. *caninum* virulence. Our previous research demonstrated that highly virulent isolates exhibit distinct expression patterns of parasite-secreted proteins, suggesting a differential regulation of effectors that may contribute to their pathogenicity [16, 17]. Among these, NcBPK1 was identified as a potential virulence factor due to its differential expression in high-virulence isolates [17]. Sequence analysis indicates that NcBPK1, like its *Toxoplasma gondii* ortholog TgBPK1 (TGME49_253330), is a predicted pseudokinase: it possesses a kinase-like fold but lacks the canonical glycine-rich loop, HRD, and DFG motifs required for catalytic activity. In *T*. *gondii*, the TgBPK1 ortholog is a pseudokinase localized to the cyst wall that plays a crucial role in tissue cyst integrity, highlighting its role in chronic infection and persistence [18]. On the basis of these findings, we hypothesized that NcBPK1 might similarly contribute to stage conversion in *N*. *caninum*, and that its deletion could impair parasite persistence, thereby modifying or altering parasite virulence.

In this study, we investigated the contribution of NcBPK1 to *N*. *caninum* virulence by generating a gene knockout (KO) strain using CRISPR/Cas9 technology. We characterized the virulence phenotype of the mutant strain in a pregnant mouse model of congenital neosporosis. The lytic cycle was also characterized in bovine monocyte-derived macrophages (BMDMs). This approach provides new insights into the role of NcBPK1 in parasite biology and host–pathogen interactions, potentially shedding light on novel targets for intervention strategies against neosporosis.

## Methods

### Generation of knockout and complemented strains

The *NcBpk1* KO strain (*Nc*Δ*BPK1)* was generated using the CRISPR/Cas9 system, following the methodology previously described [19]. In summary, guide RNAs (gRNAs) targeting both the 5′ and 3′ regions of the *NcBpk1* coding sequence (ToxoDB ID NCLIV_007770) were designed in silico. These gRNA sequences were subsequently inserted into the *Bsa*I restriction site of the pSS013-Cas9 plasmid (pU6 plasmid, Addgene #52,694) [20]. To facilitate gene disruption, the pLoxP-mCherry-DHFR plasmid (Addgene #70,147), containing the dihydrofolate reductase–thymidylate synthase (*DHFR-TS*) gene conferring pyrimethamine resistance, was used as a donor template. Tachyzoites of the Nc-Spain7 (wild-type, WT) isolate (approximately 3 × 10^7^) were electroporated with both the gRNA-containing plasmids and the *Not*I-linearized mCherry-DHFR template at a 5:1 insert-to-gRNA molar ratio. Following transfection, selection was initiated 24 h later with 10 μM pyrimethamine (Sigma-Aldrich, St. Louis, MO, USA) and maintained for at least three passages before isolating individual clones through limiting dilution.

For the generation of complemented strains, the *NcBpk1* coding region, including 1000 bp upstream and downstream of the start and stop codons, was amplified. To improve transfection efficiency, approximately 800 bp of homology arms flanking the gRNA cleavage site were added around the exogenous *NcBpk1* sequence. The amplified fragments were cloned into the multiple cloning site of the pUC19 vector, producing the pUC19-NcBPK1 plasmid. Transfections were carried out using *Kpn*I-linearized versions of these plasmids, along with a pU6 plasmid carrying a gRNA sequence targeting the 5′ region of the uracil phosphoribosyl-transferase (UPRT) gene (NCLIV_056020), at a 1:5 molar ratio (gRNA:insert). Selection was performed using 15 μM 5-fluorodeoxyuridine (FUDR, Sigma-Aldrich, St. Louis, MO, USA), and individual clones were obtained via limiting dilution.

To verify successful integration of *DHFR-TS* at the *NcBPK1* locus and confirm both gene deletion and reintroduction, Sanger sequencing and PCR analyses were conducted. Genomic DNA from isolated clones was extracted using the Maxwell® 16 Cell LEV DNA Purification Kit (Promega, Madison, WI, USA), and PCR reactions were performed with *Taq* DNA polymerase (Ecogen, Madrid, Spain) in 25 μl reaction volumes, following the manufacturer’s guidelines. The sequences of all primers used are provided in Supplementary Table S1. Additionally, NcBPK1 expression was evaluated through immunofluorescence staining (see section “Immunofluorescence Staining”).

### Validation of *DHFR-TS* gene integration by TaqMan-qPCR

To confirm the unique integration of *DHFR-TS* gene in the KO strain, we simultaneously evaluated both the number of tachyzoites corresponding to a given DNA amount and the copy number of the *DHFR-TS* DNA fragment within that same amount, as previously described [21]. Briefly, parasite quantification was performed by generating a standard curve for *N*. *caninum* on the basis of tenfold serial dilutions, ranging from 8.5 × 10^4^ to 8.5 ng/μl of tachyzoites DNA, and by amplifying the *N*. *caninum* Nc5 sequence or the *DHFR-TS* gene. Amplifications were carried out in a total volume of 10 μl using the primers and probes listed on Supplementary Table S1. From each sample, 4.25 ng or 8.5 ng of DNA were added in the reaction mix. All amplifications were conducted in triplicates.

### Parasite culture

The high-virulence *N*. *caninum* isolate Nc-Spain7 was used as the parental strain for generating the KO mutant lacking *NcBpk1*. Tachyzoites from these strains were routinely propagated in MARC-145 cell monolayers under conditions previously described [14]. Cultures were passaged every three days onto fresh cell layers to maintain parasite viability. For both in vivo and in vitro experiments, tachyzoites were collected from infected cells at low passage numbers, ensuring that most parasites remained intracellular at the time of harvest. Prior to infection assays involving bovine monocyte-derived macrophages (BMDM) and human foreskin fibroblasts (HFF), tachyzoites were purified using PD-10 Desalting Columns (Cytiva, Chicago, USA). Parasite concentration and viability were assessed using Trypan blue exclusion and quantified with a Neubauer chamber.

### Transmission electron microscopy (TEM)

Nc-Spain7 tachyzoites grown in MARC-145 monolayers were fixed in PBS containing 3% paraformaldehyde and 0.05% glutaraldehyde, embedded in LR-White resin and sections were cut on an ultramicrotome and loaded onto EM-grids as previously described [22, 23]. We obtained a rabbit polyclonal antibody specific to NcBPK1 (α-NcBPK1) from a commercial supplier (GenScript, Rijswijk, Netherlands). Unspecific binding sites were blocked in PBS-3% BSA, and the α-NcBPK1 antiserum (diluted 1:500 in PBS-0.3% BSA), was applied for 1 h in a moist chamber. After three washes in PBS, sections were incubated with goat anti-rabbit conjugated to 10 nm gold particles (Aurion, Wageningen, The Netherlands) at a dilution of 1:5 in PBS-0.3% BSA. The samples were washed in PBS again, rinsed once with water and air-dried. They were stained with Uranyless® and lead citrate (Electron Microscopy Sciences, Hatfield PA, USA), and imaging of the specimens was performed on a FEI Morgagni TEM at 80 kV equipped with a Morada digital camera system (12 Megapixel) operating at 80 kV.

### Evaluation of *Nc*Δ*BPK1* virulence in a BALB/c murine model of congenital and cerebral neosporosis

The virulence of *NcBpk1* KO parasites was assessed using the established BALB/c mouse model for congenital and cerebral neosporosis [24]. Female mice were obtained from Janvier Labs (Laval, France) and randomly distributed in four groups (20 mice per group). Animals were housed under standard conditions with a controlled light/dark cycle and provided with food and water ad libitum. All mice underwent a 15-day acclimatization period before the start of the experiments.

Pregnancy was induced through oestrus synchronization using the Whitten effect [25], in which two females are housed with one male for three days of controlled mating. Day 0 of pregnancy is defined as the first day of cohabitation. Subsequently, all females were randomly assigned to the experimental groups and subcutaneously inoculated at mid-gestation (gestational days 7–10) with 10^5^ tachyzoites per mouse from either knockout (*Nc*Δ*BPK1*) or complemented (*Nc*Δ*BPK1::BPK1*) strains, the wild-type Nc-Spain7 isolate (WT), or left uninfected (PBS control). Pregnancy was later confirmed between days 15 and 18 post-mating by weight monitoring, after which pregnant females were housed individually for delivery.

Clinical signs were monitored daily from birth until 30 days postpartum (pp) in dams and their offspring. Clinical manifestations indicative of murine neosporosis were scored based on a severity scale ranging following the criteria established previously [26]: 0 (normal), 1 (ruffled fur), 2 (hunched posture), 3 (severe weight loss), and 4 (neurological symptoms). As a humane endpoint, animals experiencing ≥ 20% body weight loss and neurological signs were euthanized to prevent unnecessary suffering. Nonpregnant mice were euthanized at 30 days postinfection (pi), while dams and their offspring were euthanized at 30 days postpartum (pp). Euthanasia was performed using CO₂ inhalation followed by cervical dislocation. Brain and serum samples from all subjects were collected and stored at −80 °C for subsequent analysis.

For the congenital *N*. *caninum* model, reproductive parameters including fertility rate (percentage of pregnant mice), litter size (number of pups per dam) and neonatal mortality (deaths occurring from day 2 to day 30 pp) were recorded. The cerebral neosporosis model was assessed in dams at the chronic infection stage (30 days pp) by quantifying brain parasite burden using qPCR (see section “DNA Extraction and qPCR Parasite Quantification”). Additionally, serum samples collected at 30 days pp from infected mice were tested by ELISA to measure *N*. *caninum*-specific IgG1 and IgG2 levels, as described below.

### Proliferation dynamics of *N*. *caninum* in naïve BMDM

BMDMs were isolated from the peripheral blood of a healthy adult cow following the protocol previously described [15]. In brief, peripheral blood mononuclear cells (PBMCs) were separated using density gradient centrifugation with Histopaque 1077 (Sigma-Aldrich, St. Louis, MO, USA). Monocytes were then purified using mouse anti-human CD14 antibodies conjugated to microbeads (Miltenyi Biotec Ltd., San Diego, CA, USA), according to the manufacturer’s guidelines. Isolated monocytes were plated in 6-well plates at a density of 3 × 10^6^ cells per well and cultured in RPMI 1640 medium (Sigma-Aldrich, St. Louis, MO, USA) supplemented with 10% heat-inactivated fetal calf serum (FCS), 50 μg/ml of gentamicin, 2 mM of L-glutamine, 50 μM of β-mercaptoethanol, and 20 mM of HEPES (all from Thermo Fisher Scientific). Additionally, 100 ng/ml of GM-CSF (Kingfisher Biotech Inc., St. Paul, MN, USA) was included to promote differentiation into BMDMs. After 5 days of incubation, the differentiated BMDMs were harvested and reseeded at densities of either 3 × 10^6^ cells per well in 6-well plates or 3 × 10^5^ cells per well in 24-well plates.

To evaluate the proliferation kinetics of *Nc*Δ*BPK1* and the wild-type Nc-Spain7 strain, the number of tachyzoites within BMDMs was quantified at specific time points using qPCR. For this purpose, infections were initiated 24 h post-seeding to minimize macrophage stress induced by harvesting. Next, BMDM cultures were infected with highly viable parasites (purified from MARC-145 cells within 1 h) at a multiplicity of infection (MOI) of 0.5:1. Uninfected BMDMs served as negative controls. Cells were lysed at defined intervals during the *N*. *caninum* lytic cycle (8, 24, 36, 48, 60, and 72 h pi) by adding a buffer solution containing 200 μl PBS, 180 μl lysis buffer (Qiagen, Germany), and 20 μl proteinase K (Qiagen, Germany). The lysates were then transferred to DNAse-free tubes (1.5 ml) and stored at −80 °C until DNA extraction for parasite quantification via qPCR (see section “DNA Extraction and qPCR Parasite Quantification”). All experiments were conducted using six replicates from two independent trials (12 replicates per condition), spaced at least two weeks apart.

In parallel, BMDMs grown on round glass coverslips were infected under identical conditions and fixed at the same time points of the lytic cycle. Single immunostaining (described below) was performed to assess parasite proliferation microscopically. At 48 h pi, when tachyzoites were still predominantly intracellular, vacuole size and cell infection rate (cIR; percentage of infected cells containing at least one tachyzoite) were determined by immunofluorescence (see section “Immunofluorescence Staining”). For each coverslip, at least five fields were analyzed.

### Effect of IFN-γ on *N*. *caninum* growth in BMDM

BMDMs were plated in 24-well culture plates and exposed to varying concentrations of IFN-γ (0.1 and 10 ng/ml; Kingfisher Biotech Inc., St. Paul, MN, USA) four hours after seeding. Following a 24 h stimulation period, cells were infected with tachyzoites from the Nc-Spain7 and *Nc*Δ*BPK1* strains at a MOI of 0.5. At 48 and 60 h pi, samples were harvested using a lysis solution as detailed above (see “Proliferation dynamics of *N*. *caninum* in naïve BMDM”) and stored at −80 °C for subsequent DNA extraction. Tachyzoite proliferation was quantified through qPCR and expressed as relative growth (%) compared with the IFN-γ untreated sample. All conditions were tested using six replicates in two independent experiments (12 replicates per condition).

### DNA extraction and qPCR parasite quantification

To assess parasite loads in the brain of infected dams, DNA was extracted from 50 to 100 mg of tissue using the Maxwell^®^ 16 Mouse Tail DNA Purification Kit (Promega, Madison, WI, USA). DNA concentration was measured via spectrophotometry using a Nanophotometer^®^ (Implen GmbH, Munich, Germany). For the analysis of the proliferation kinetics of the KO mutant strains in BMDMs, DNA extraction was performed with the DNeasy Blood & Tissue Kit (Qiagen, Germany) according to the manufacturer’s instructions.

Parasite quantification was carried out using the 7500 FAST Real-Time PCR System (Applied Biosystems, Foster City, CA, USA). The Nc5 region was used to quantify *N*. *caninum* DNA, while 28S rRNA gene was employed to quantify host DNA in mouse [27]. Parasite burden was calculated by interpolating the cycle threshold (Ct) values against a standard curve derived from tachyzoite counts ranging from 10^−1^ to 10^5^, followed by normalization to host DNA levels. All primers used are listed in Supplementary Table S1.

### Immunofluorescence staining

To assess the presence of NcBPK1 protein in the KO and complemented strains, immunofluorescence was conducted following the protocol previously described [28]. Infected MARC-145 cells were washed three times with PBS and fixed in ice-cold methanol for 10 min. The cells were then blocked and permeabilized in PBS containing 3% BSA and 0.25% Triton-X 100 for 45 min at 37 °C. The cultures were incubated with the monoclonal antibody α-NcSAG1 (1:250) to detect surface markers [29] and polyclonal antibody α-NcBPK1 (1:100) (GenScript, Rijswijk, Netherlands) for 1 h at 37 °C. Secondary antibodies, Alexa Fluor 594-conjugated goat anti-mouse IgG and Alexa Fluor 488-conjugated goat anti-rabbit IgG (Life Technologies, Carlsbad, CA, USA), were used at a dilution of 1:1000 for 1 h at 37° C. Nuclei were stained with DAPI (1:10,000).

For infected BMDMs, a double immunofluorescence protocol was followed. The cultures were initially fixed using 0.05% glutaraldehyde and 3% paraformaldehyde. After permeabilization with Triton X-100, cells were incubated with polyclonal rabbit anti-*N*. *caninum* serum (1:2,000) [30] for 1 h at 37 °C, followed by incubation with Alexa Fluor 488-conjugated goat anti-rabbit IgG (1:750) for 1 h at 37 °C. To stain macrophages, Alexa Fluor-594 Phalloidin (Life Technologies, Carlsbad, CA, USA) was applied for 30 min at 37° C. Nuclei were stained with DAPI at a dilution of 1:10,000 in PBS.

Images were captured using an inverted fluorescence microscope (Nikon Eclipse TE200) at 40 × or 100 × magnification, and data were processed using NIS Elements Imaging Software (v. 5.30.04).

### Assessment of humoral immune response in murine *N*. *caninum* infections

The levels of *N*. *caninum*-specific IgG1 and IgG2a in serum samples from female mice were quantified using an ELISA assay, as previously described [24]. In brief, soluble tachyzoite antigen (0.125 μg/well) was coated onto 96-well plates, and serum samples were diluted 1:100 before analysis. Peroxidase-conjugated anti-mouse IgG1 or IgG2a (1:5,000; Southern Biotechnology, Birmingham, AL, USA) was used as secondary antibodies. Control sera included samples from previous studies where mice were either experimentally infected with Nc-Spain7 or left uninfected [31]. Absorbance was measured at 405 nm, and the results were expressed as a relative index percentage (RIPC) using the formula: RIPC = (OD sample—OD negative control)/(OD positive control—OD negative control) × 100.

### Tachyzoite to bradyzoite stage conversion assay

To evaluate potential defects in bradyzoite formation in the KO strains, conversion assays were performed using *N*. *caninum* tachyzoites cultured in HFF. HFF monolayers were seeded in 6-well plates at a density of 3 × 10^6^ cells per well and infected at a MOI of 1. Bradyzoite differentiation was induced by adding 70 μM sodium nitroprusside at 24 h pi, and maintained throughout the seven-day experiment, following previously established protocols [32]. Total RNA was extracted, and complementary DNA (cDNA) was synthesized from 50 ng of RNA using the NZY First-Strand cDNA Synthesis Kit (Nzytech, Portugal) according to the manufacturer’s instructions. Expression levels of *NcSag4* (bradyzoite marker) and *NcSag1* (tachyzoite marker) and *NcBpk1* were quantified by RT-qPCR using specific primers (Supplementary Table S1). The reactions were performed on a 7500 FAST Real-Time PCR System (Applied Biosystems, Foster City, CA, USA) with the GoTaq® qPCR Master Mix (Promega, Madison, WI, USA). All samples were processed in duplicate, and results were expressed as fold induction calculated using the 2^−ΔΔCt^ method (Schmittgen and Livak, 2008). The ΔΔCt values were obtained by normalizing *NcSag4* and *NcSag1* cycle threshold (Ct) values to Nc18S rRNA [33] and comparing them to unstressed control parasites from each time point.

### Statistical analysis

Mortality rates were assessed using Fisher’s test, while survival analysis was performed using the Kaplan–Meier method to estimate survival percentages at different time points. The log-rank (Mantel-Cox) test was applied to compare survival curves and determine median survival times. Clinical scores and parasite burdens across groups were analyzed using the Kruskal–Wallis test followed by Dunn’s multiple-comparison test. Litter size and antibody levels were compared using one-way ANOVA with Tukey’s post hoc test, following assessment of normal distribution with the D’Agostino-Pearson test. For parameters such as cell infection rate (cIR), parasitic vacuole size, proliferation kinetics, and mRNA expression levels, a parametric one-way ANOVA followed by Dunnett’s test for multiple comparisons was used to compare all groups to the WT-infected group. Statistical significance was set at *p* < 0.05 for all analyses. Data were analyzed using GraphPad Prism v.7.0 (GraphPad Software, San Diego, CA, USA).

## Results

### Generation and validation of *NcBpk1* knockout and complemented strains

**Fig. 1:**
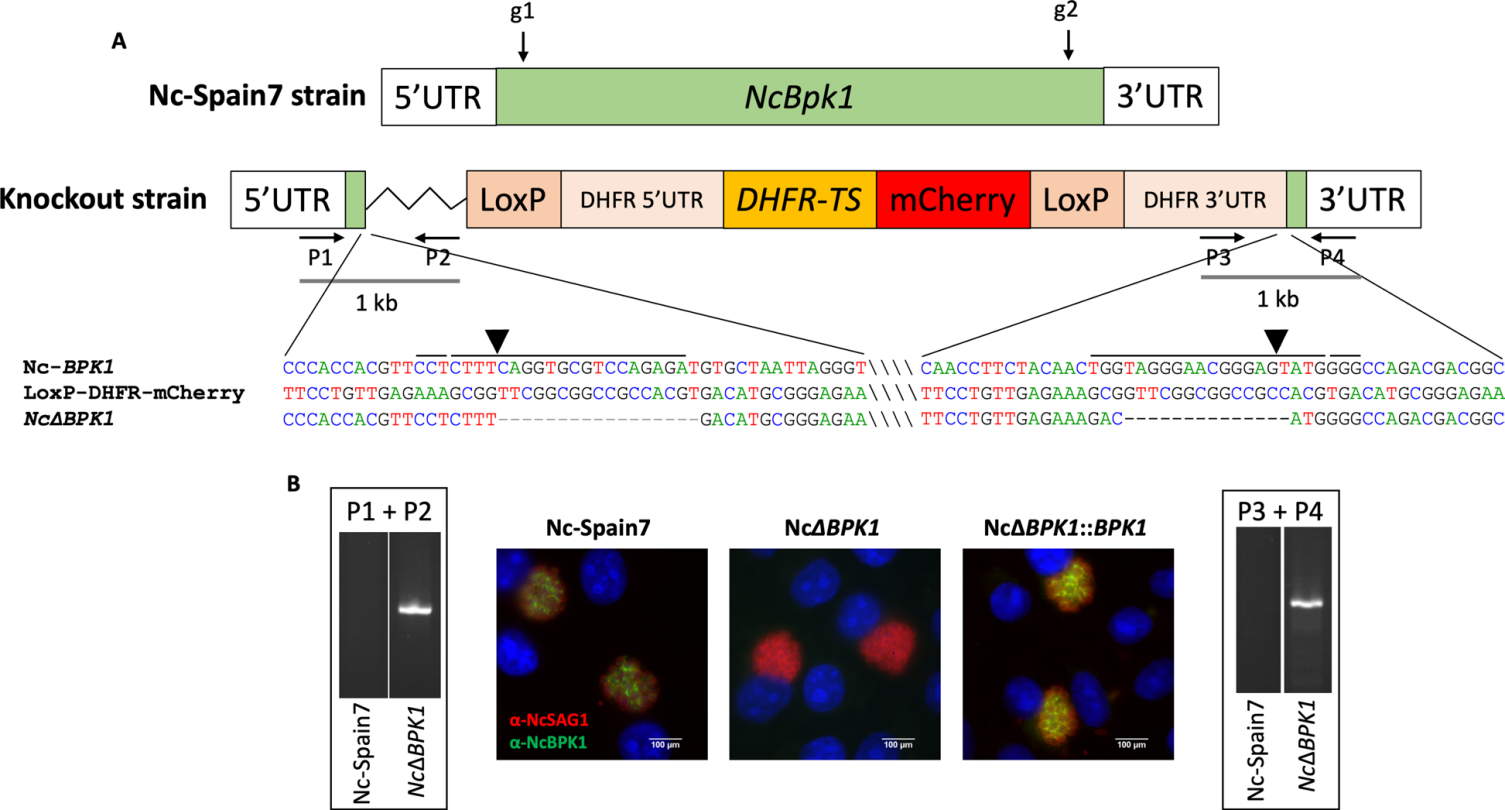
Generation of the NcBPK1 KO strain (*Nc*Δ*BPK1*) in *N*. *caninum*. **A** The schematic illustrates the *Nc*Δ*BPK1* mutant clone, highlighting the integration of the donor template. Arrows indicate the position and orientation of primers (P1–P4) used in diagnostic PCR. Successful integration into the *NcBPK1* locus was confirmed by PCR amplification using primer pairs P1 + P2 and P3 + P4. Sequence alignments display the regions flanking the CRISPR cutting site, comparing the KO clone with the wild-type *NcBPK1* locus and the loxP-DHFR-mCherry plasmid. Guide RNA (gRNA, g1, and g2) target sites and protospacer adjacent motif (PAM) sequences are marked above the sequences, while slashes separate non-adjacent regions for clarity. Arrowheads indicate CRISPR cutting sites. Abbreviations: UTR, untranslated region; *DHFR-TS*, dihydrofolate reductase-thymidylate synthase. **B** For immunofluorescence analysis, MARC-145 cells infected with different *N*. *caninum* strains (Nc-Spain7, *Nc*Δ*BPK1* and *Nc*Δ*BPK1::BPK1*) were stained to visualize nuclei (DAPI, blue), parasite surfaces (NcSAG1, red), and NcBPK1 protein (green)

To investigate the function of NcBPK1, we successfully generated an *Nc*Δ*BPK1* strain by completely replacing the *NcBpk1* coding sequence with a *DHFR-TS* selection cassette. The deletion was confirmed through PCR using primers specific to the *NcBpk1* flanking regions and the *DHFR-TS* cassette (Supplementary Table S1). In *Nc*Δ*BPK1* parasites, the expected PCR amplicons were detected, while they were absent in the parental strain (Fig. 1A). Further validation was performed by Sanger sequencing, and the loss of NcBPK1 protein expression was assessed through immunofluorescence analysis (Fig. 1B).

To generate a complemented strain, we optimized the existing complementation method by incorporating flanking homologous regions to enhance recombination efficiency. The *NcBpk1* gene was reintroduced into the UPRT locus through double homologous recombination, and single clones were obtained by limiting dilution. Immunofluorescence staining confirmed the expression of NcBPK1 protein in the complemented strain, showing levels comparable to those of the Nc-Spain7 wild-type strain (Fig. 1B). These findings confirmed the successful construction of the *Nc*Δ*BPK1* knockout strain and its corresponding complemented version, *Nc*Δ*BPK1::BPK1*.

### Confirmation of single-copy integration of the *DHFR-TS* cassette in *Nc*Δ*BPK1*

The *N. caninum Nc*Δ*BPK1* strain was analyzed using the duplex TaqMan qPCR assay previously described [21]. For *N*. *caninum* WT parasites, the number of tachyzoites calculated via *DHFR* amplification matched that obtained through Nc5 sequence amplification, resulting in a ratio of 1. For a KO strain with a single copy insertion of the *DHFR-TS* cassette, the *DHFR*-based quantification resulted in twice the number of tachyzoites compared with Nc5-based quantification, leading to a ratio of 2. In the case of multiple insertions, this ratio would have been greater than 2.

**Fig. 2:**
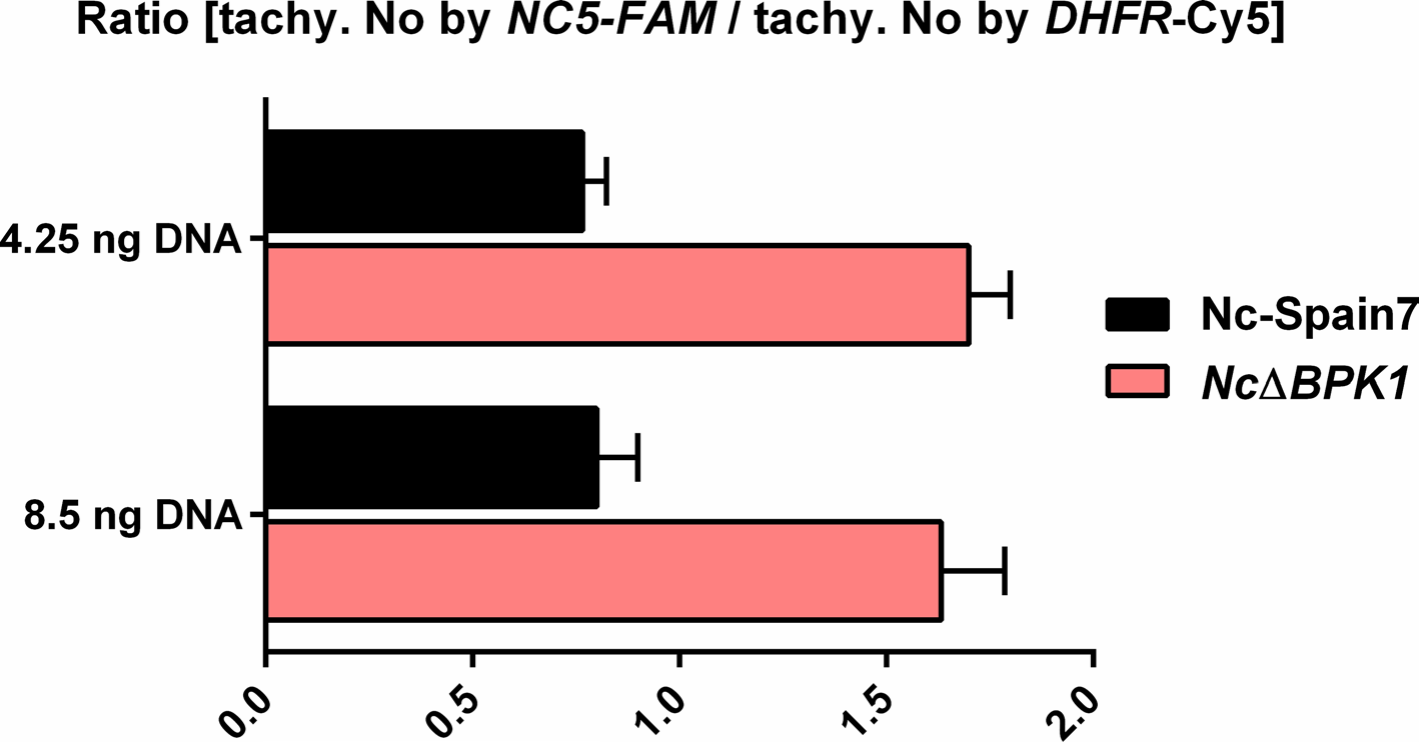
Quantification of *DHFR-TS* gene copy number in the *Nc*Δ*BPK1* strain by TaqMan qPCR. The copy number of the integrated *DHFR-TS* cassette was obtained from the ration between tachyzoite counts estimated using *DHFR* and Nc5 primers, using two different DNA concentrations. Error bars represent the standard deviation from triplicate reactions for each sample

As observed in Fig. 2, the number of WT tachyzoites determined by Nc5 amplification and *DHFR* amplification was similar for both tested DNA quantities, resulting in a ratio of 0.8. For the *Nc*Δ*BPK1* strain, the number of tachyzoites determined by *DHFR* quantification was twice the number of tachyzoites determined by Nc5 quantification, thus resulting in a ratio of 1.6 for both quantities. This ratio was twice the ratio found in the WT (0.8), indicating a single insertion of the *DHFR-TS* cassette, therefore confirming the unique integration of the selectable marker within the genome.

### Immunogold electron microscopy of *N*. *caninum* tachyzoites localized NcBPK1 in the rhoptry matrix

**Fig. 3:**
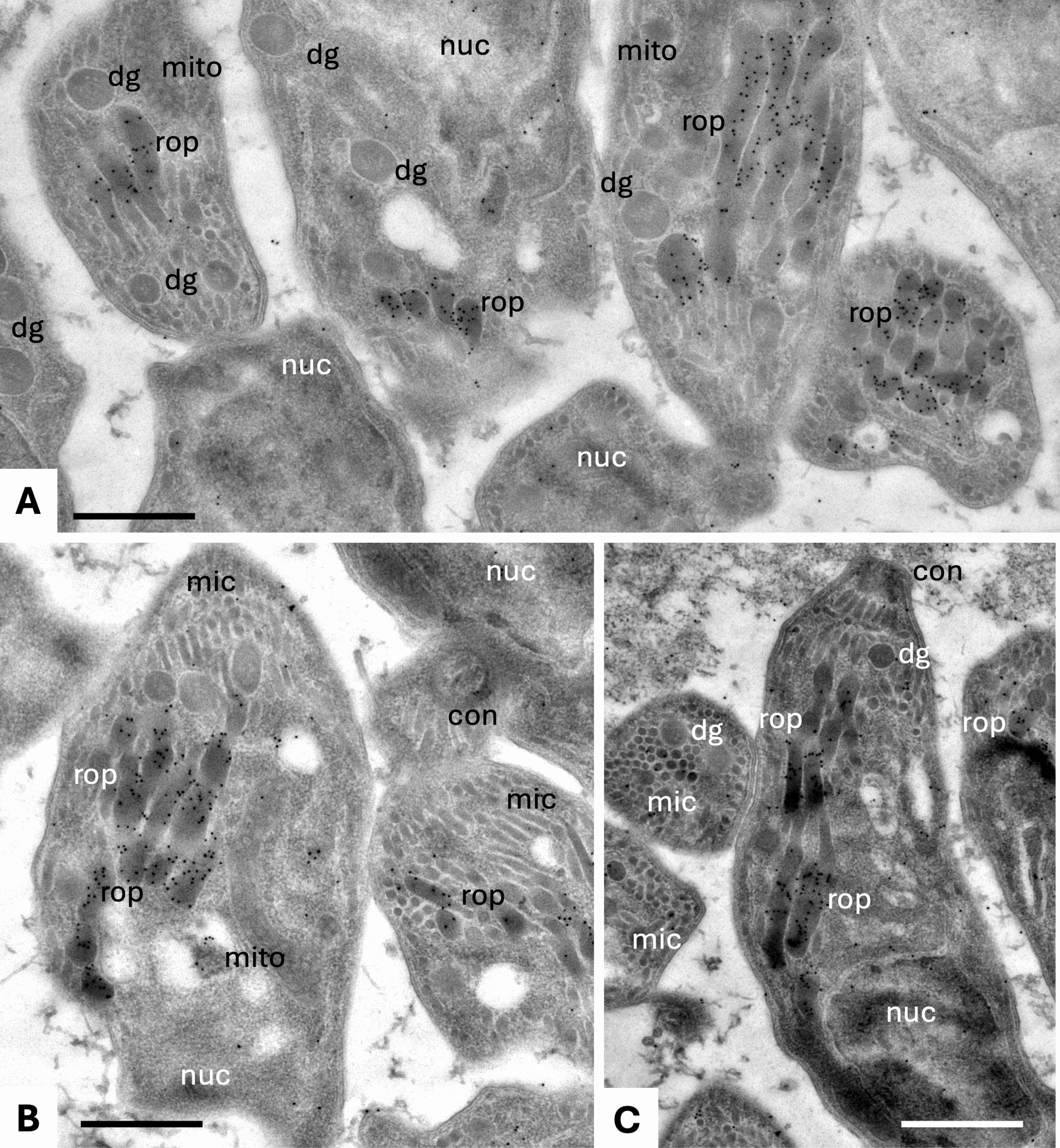
Immunogold-transmission electron microscopy of *N*. *caninum* tachyzoites cultured in MARC-145 cells, highlighting NcBPK1 localization. Parasites are localized within a parasitophorous vacuole. Panel A, B and C correspond to different representative images of parasites, illustrating the specific localisation pattern. 10 nm gold particles indicate the localization of NcBPK1, which is predominantly found in the rhoptry matrix, but not in other parasite organelles or structures; rop = rhoptries; dg = dense granules; mic = micronemes; con = conoid; nuc = nucleus; mito = mitochondrion. Bar in A and B = 1 µm; C = 1.3 µm

On-section immune-gold labeling was performed to study the localization of NcBPK1 in *N*. *caninum* tachyzoites (Fig. 3). In LR-White embedded specimens, parasite structures reminiscent for apicomplexans such as the apical conoid, secretory organelles including rhoptries, micronemes and dense granules, and the nucleus, were clearly identifiable. The vast majority of gold particles was found to be associated with the matrix of the parasite rhoptries, which are one of the three types of secretory organelles found in apicomplexan parasites (Fig. 3). Since secretory products originating from rhoptries are implicated in host cell invasion and intracellular host–parasite interactions, this suggests that NcBPK1contributes to rhoptry-mediated functions during lytic cycle.

### Deletion of *NcBpk1* exacerbates parasite virulence in mice

**Fig. 4:**
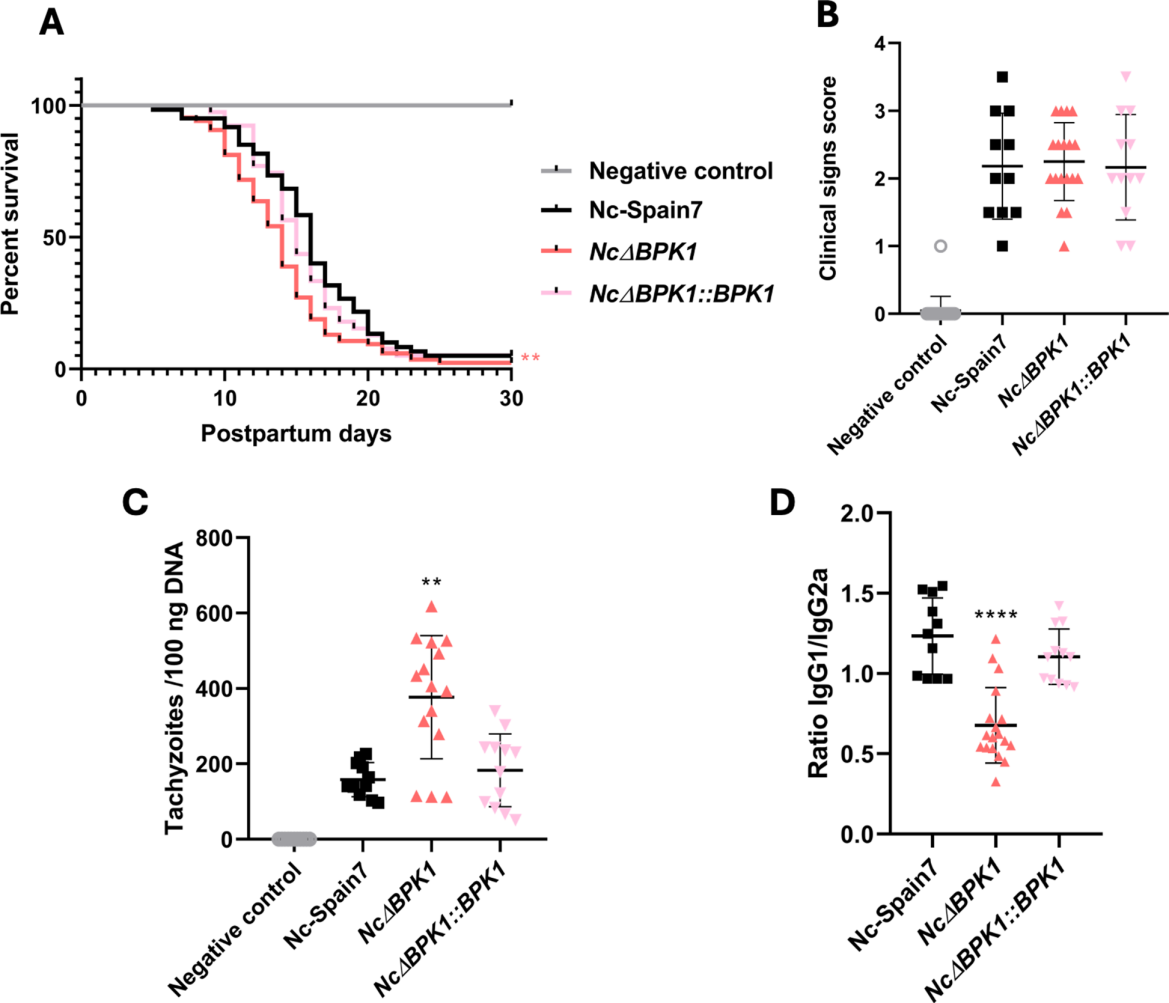
Impact of Nc-Spain7, *Nc*Δ*BPK1* and* Nc*Δ*BPK1::BPK1* infection in a BALB/c murine model of neosporosis. **A** Kaplan–Meier survival curve of offspring from pregnant mice infected with different strains of *N*. *caninum*. Dams were inoculated with 10^5^ tachyzoites on day 7 of gestation, and pup survival was monitored until day 30 pp. Each step in the curve represents a mortality event. Endpoint survival proportions at day 30 did not differ significantly between infected groups (Fisher’s exact test). However, pups from *Nc*Δ*BPK1*-infected dams showed a significantly shorter median survival time compared with the WT group (Log-rank test, *p* < 0.01). Asterisks indicate statistical significance for the Kaplan–Meier comparison of survival kinetics. **B**: Clinical signs recorded in dams infected with different strains of *N*. *caninum*. Signs were scored based on severity (0: no symptoms; 1: ruffled fur; 2: hunched posture; 3: severe weight loss; 4: neurological impairment). Each dot represents an individual mouse. Statistical analysis was performed relative to the Nc-Spain7 group, and no differences were observed between groups. **C**: Brain parasite burden (number of parasites per 100 μg of host DNA) in dams infected with different *N*. *caninum* strains. Each dot represents an individual value, with median and standard deviation shown as horizontal and vertical lines, respectively. Significant differences between infected groups are marked by asterisks (*p* < 0.01; Kruskal–Wallis test, Dunn’s post-test). **D**: *Neospora*-specific IgG1/IgG2a antibody ratio at 30 days pi in dams. Each dot represents an individual value, with median and standard deviation shown as horizontal and vertical lines, respectively. Significant differences between groups are indicated by asterisks (*p* < 0.0001; one-way ANOVA, Tukey’s post hoc test)

To assess the impact of NcBPK1 on parasite virulence, pregnant and non-pregnant mice were infected with the *Nc*Δ*BPK1* strain, as well as the WT (Nc-Spain7) and complemented (*Nc*Δ*BPK1::BPK1*) strains. Neonatal mortality rates were close to 100% across all infected groups, with no significant differences between the *Nc*Δ*BPK1* and WT infections (*p* > 0.05, Fisher’s exact test). Pups in the *Nc*Δ*BPK1-*infected group had a significantly shorter median survival time (14 days) compared with those infected with the WT strain (16 days) (*p* < 0.05, Log-rank test) (Table 1 and Fig. 4A). In addition, complementation of the *NcBpk1* gene into the *Nc*Δ*BPK1* strain reduced virulence to levels comparable to the WT strain (*p* > 0.05, Log-rank test) (Table 1 and Fig. 4A). This suggests that loss of NcBPK1 is associated with an increase in parasite virulence in the mouse model, as reflected by reduced survival time of the offspring. Statistical analysis revealed no significant differences among the groups in terms of pregnancy rates (*p* > 0.05, Fisher’s exact test) or litter size (*p* > 0.05, one-way ANOVA) (Table 1).

**Table 1:** Effects of *Neospora caninum *infection on pregnant BALB/c mice and their offspring

Group	Fertility (%)^a^	Litter size^b^	Postnatal survival (%)^e^	Median survival time (days)^f^
Negative control	11/14 (78)	4.10 ± 2.11	43/43 (100)	> 30
Nc-Spain7	11/18 (61)	3.00 ± 1.54	2/28 (7)	16
*Nc*∆*BPK1*	18/20 (90)	5.17 ± 1.61	2/85 (2)	14
*Nc*∆*BPK1::BPK1*	12/18 (67)	4.25 ± 2.05	2/37 (5)	15

^a^Percentage of pregnant mice per group. ^b^Number of pups delivered per dam (mean ± standard deviation). ^c^Percentage of pups surviving at day 30 postpartum. ^d^Postpartum day at which 50% mortality was observed

Clinical manifestations began to appear in all challenged dams during the second week pi, progressing from mild clinical signs such as ruffled fur and lethargy to more severe signs, including anorexia, inactivity and neurological impairment. No significant differences in clinical signs were observed between groups (*p* > 0.05, Kruskal–Wallis, Dunn’s post hoc test) (Fig. 4B). Notably, mice infected with *Nc*Δ*BPK1* had significantly higher parasite burdens in the brain compared with those infected with WT or *Nc*Δ*BPK1::BPK1* (*p* < 0.01, Kruskal–Wallis, Dunn’s post hoc test) (Fig. 4C).

Serological analyses confirmed infection across all challenged groups, with significantly elevated IgG2a antibody levels relative to uninfected controls (*p* < 0.0001, one-way ANOVA, Tukey’s post hoc test) (data not shown). Mice infected with *Nc*Δ*BPK1* exhibited a significantly lower IgG1/IgG2a ratio (*p* < 0.0001, one-way ANOVA, Tukey’s post hoc test) (Fig. 4D). This ratio remained unchanged in mice infected with either the WT or the complemented strain (Fig. 4D).

### NcBpk1 deletion enhances *N*. *caninum* proliferation in BMDM

**Fig. 5:**
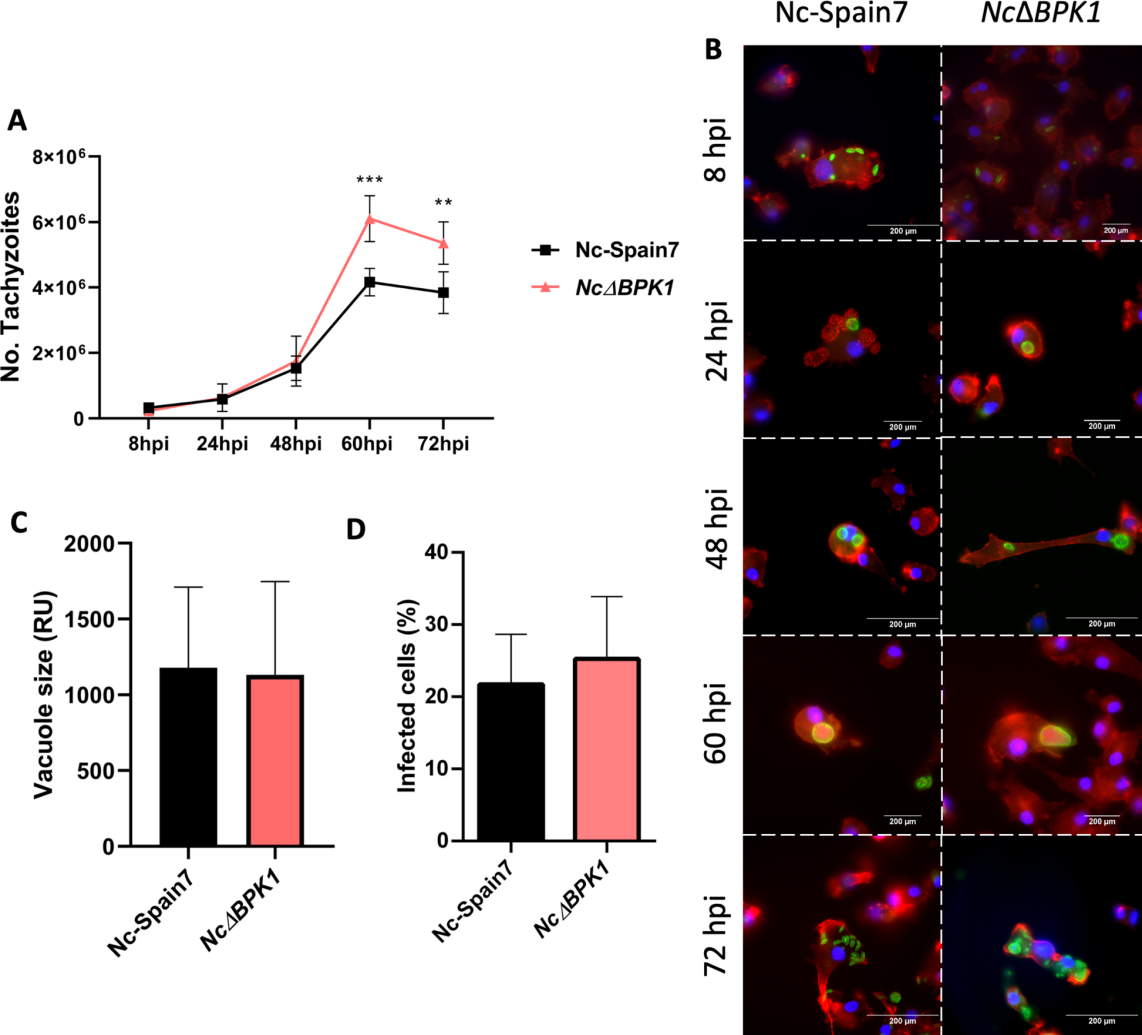
Characterization of Nc-Spain7 and *Nc*Δ*BPK1* proliferation and infection dynamics in BMDMs. **A**: Growth kinetics of the parental Nc-Spain7 and knockout *Nc*Δ*BPK1* strains in BMDMs over time by qPCR analysis. The graph illustrates the parasite proliferation using a multiplicity of infection (MOI) of 0.5:1. Error bars represent the standard deviation (SD). Data were collected from 12 replicates across two independent experiments. Significant differences between the knockout and the Nc-Spain7 strains are indicated by asterisks (**: *p* < 0.001, one-way ANOVA, Dunnett’s post hoc test). **B**: Immunofluorescence images depicting the lytic cycle progression of Nc-Spain7 and the *Nc*Δ*BPK1* strains from 8 to 72 h pi (hpi). F-actin (red), nuclei (blue), and parasites (green) are shown. **C**: Quantification of parasitophorous vacuole size at 48 h pi, assessed through immunofluorescence staining and analyzed with NIS Elements Imaging Software. No significant differences were observed among groups. **D**: Percentage of infected BMDMs for each strain, calculated as the number of infected BMDMs relative to the total BMDMs count. Error bars represent the SD. No significant differences were observed among groups

To assess how the deletion of *NcBpk1* affects parasite fitness in the context of innate immune responses, we infected BMDMs with either the *Nc*Δ*BPK1* or the WT strain and tracked their replication dynamics over time by qPCR. Both strains showed comparable exponential proliferation rates up to 48 h pi (Fig. 5A). After this, a significant increase in parasite burden was detected in *Nc*Δ*BPK1*-infected BMDMs relative to those infected with the WT strain (*p* < 0.001, one-way ANOVA, Dunnett’s post hoc test), suggesting enhanced replication and faster expansion of the mutant line.

Immunofluorescence analyses employing a polyclonal anti-*N*. *caninum* antiserum and fluorescently coupled phalloidin to stain the macrophage F-actin filaments revealed that both strains had successfully invaded BMDMs at 8 h pi, and formed parasitophorous vacuoles (PVs) (Fig. 5B). Parasite replication commenced at 24 h pi, and PVs reached their maximum size between 48 and 60 h pi. At 48 h pi, there was no significant difference in PV size or percentage of infected cells between strains (Fig. 5C and D). By 72 h pi, host cell lysis and tachyzoite release were observed for both strains, indicating completion of the lytic cycle.

### *Nc*Δ*BPK1* and Nc-Spain7 exhibit similar susceptibility to IFN-γ-mediated growth inhibition

**Fig. 6:**
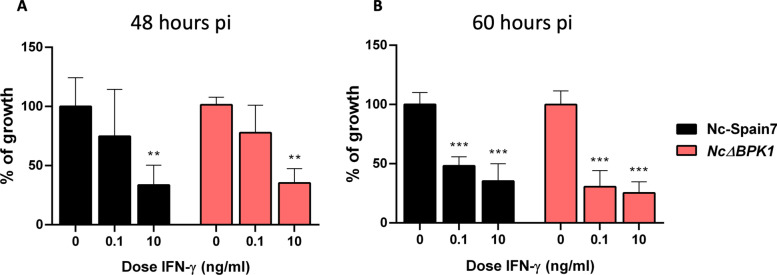
Growth inhibition of Nc-Spain7 and *Nc*Δ*BPK1* in BMDMs under IFN-γ stimulation at 48 (**A**) and 60 h pi (**B**). The bar graph depicts the parasite burden in BMDMs infected with either the parental Nc-Spain7 strain (black) or the *Nc*Δ*BPK1* strain (pink), under unstimulated conditions or increasing doses of IFN-γ (from 0.1 to 10 ng/ml). BMDMs were obtained from at least two independent experiments, with 12 replicates analyzed per condition. Error bars represent the standard deviation (SD). Significant differences compared with naïve BMDMs (non-stimulated with IFN-γ) are indicated by asterisks (**: *p* < 0.01; ***: *p* < 0.001, one-way ANOVA, Dunnett’s post-test)

To investigate the role of NcBPK1 in parasite survival under immune pressure, we assessed the proliferation of *Nc*Δ*BPK1* and WT in BMDMs treated with increasing concentrations of IFN-γ. At 48 h pi, both WT and *Nc*Δ*BPK1* exhibited a similar dose-dependent reduction in parasite growth, with significant inhibition observed at 10 ng/ml of IFN-γ (Fig. 6A) (*p* < 0.01, one-way ANOVA test, Dunnett’s comparison post hoc test). However, at 60 h pi, both strains exhibited substantial inhibition even at the lowest IFN-γ concentrations, with parasite proliferation being reduced by approximately 60% for both strains (Fig. 6B) (*p* < 0.001, one-way ANOVA test, Dunnett’s comparison post hoc test). These results indicate that the deletion of *NcBpk1* did not confer resistance to IFN-γ-mediated restriction and that both strains were similarly affected.

### Deletion of* NcBpk1* impairs the expression of bradyzoite-related genes

To determine whether the increased virulence and proliferation rate observed in the *Nc*Δ*BPK1* strain was associated with alterations in bradyzoite conversion, we performed a tachyzoite-to-bradyzoite conversion assay to assess the parasite ability to undergo stage conversion under stress conditions (sodium nitroprusside stimulation) for 1, 3, 5, and 7 days. During this period, we analyzed the expression of *NcSag4,* a well-characterized bradyzoite-specific gene in *N*. *caninum* [32].

**Fig. 7:**
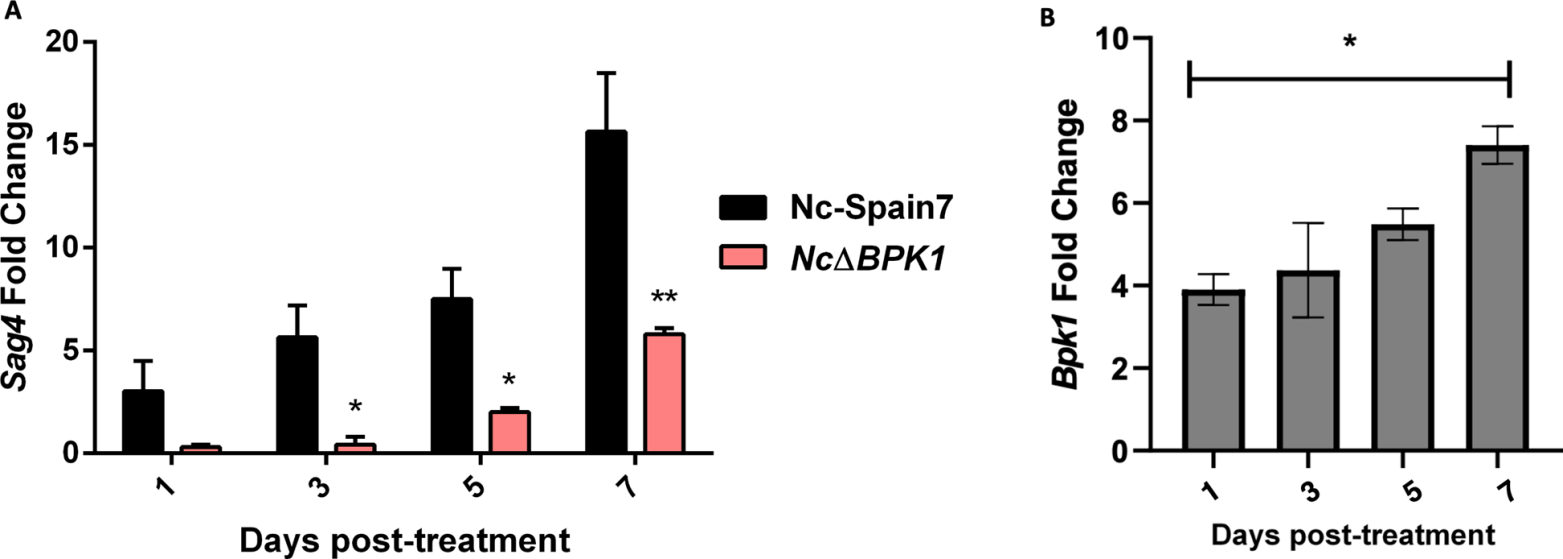
Expression levels of *NcBpk1* (**A**) and the bradyzoite-specific gene *NcSag4* (**B**) during stage conversion. Gene expression was assessed by RT-qPCR at different time points following sodium nitroprusside stimulation to induce bradyzoite differentiation. Fold change was calculated relative to untreated parasites. Error bars represent the standard deviation. Significant differences between groups at same time point are marked asterisks (*: *p* < 0.05; **: *p* < 0.01; Fisher’s F test)

Over the course of the assay, *NcSag4* transcript levels progressively increased in all strains, indicating differentiation into a bradyzoite-like stage (Fig. 7a). However, the *Nc*Δ*BPK1* strain exhibited a significantly lower and delayed upregulation of *NcSag4* expression compared with the parental WT strain. Concurrently, the expression of *NcSag1* transcripts, coding for a tachyzoite-specific surface antigen [34], remained stable throughout the assay, with no significant differences between groups (data not shown). This suggested that *Nc*Δ*BPK1* displayed a reduced ability to transition into the bradyzoite stage under stress conditions. This characteristic could help explain the increased virulence observed in the mutant.

To further investigate whether NcBPK1 might contribute to the transition into the bradyzoite stage under stress conditions, we measured its transcript levels during the conversion assay (Fig. 7b). *NcBpk1* transcripts were already detectable at day 1, corresponding to the tachyzoite stage, but gradually increased as differentiation progressed, reaching their highest levels at day 7. This expression pattern suggests that NcBPK1 is not restricted to the proliferative tachyzoite stage, but rather becomes upregulated during differentiation, supporting a possible role in stage conversion or in the persistence of chronic infection.

## Discussion

On the basis of transcriptomic and proteomic analysis comparing tachyzoites of *N*. *caninum* isolates with different virulence profiles, NcBPK1 was identified as a key protein up-regulated in highly virulent strains in different environments, such as bovine macrophages or trophoblast cells, and in different stages of the parasite lytic cycle [16, 17, 41–43]. In contrast, its orthologue in *T*. *gondii*, TgBPK1, is a known component of the cyst wall and is essential for cyst development and infection in vivo [35]. Despite containing a kinase fold, TgBPK1 lacks the conserved catalytic residues required for enzymatic activity [18]. Like many pseudokinases, TgBPK1 is thought to function as a molecular scaffold, regulating active kinases and signaling pathways by facilitating the assembly of multiprotein complexes [36]. Pseudokinases have been extensively studied in *T*. *gondii* and are recognized as key regulators of host cell signaling [37] often promoting phosphorylation or activation of other proteins. Several rhoptry proteins in *T*. *gondii*, including TgROP16, TgROP38 and TgROP5, have been shown to influence host cell signaling pathways and transcriptional responses through phosphorylation-dependent mechanisms [38–40]. Although previous studies failed to detect BPK1 within intracellular parasites [35], our results demonstrated that NcBPK1 localizes within the rhoptry matrix. This subcellular localization suggests a potential role in signaling or regulatory processes.

The increased expression of NcBPK1 in virulent *N*. *caninum* isolates likely reflects its importance in parasite pathogenicity favoring either replication or persistence depending on host conditions. However, deletion of this gene, resulted in increased virulence, as evidenced by the reduced neonatal survival times in *Nc*Δ*BPK1-*infected mice compared with the WT strain. Similarly, dams infected with *Nc*Δ*BPK1* exhibited significantly higher parasite loads in the brain than those infected with the WT strain. This further supported the role of NcBPK1 in parasite virulence, given that elevated brain parasite burdens are a well-established indicator of *N*. *caninum* strain pathogenicity [44]. These in vivo data were obtained using a well-established and robust pregnant mouse model of congenital neosporosis, which has been extensively applied [24, 31, 44–48]. The use of this standardized model, together with infection by the same parasite isolate (Nc-Spain7), minimizes variability and provides reproducible outcomes across experiments, allowing reliable comparisons of phenotypic differences between strains.

To date, no studies have specifically investigated the role of NcBPK1 by generating KO-mutants. However, a similar phenotype to the one observed for *NcΔBPK1* has been described in *T*. *gondii* KO mutants targeting bradyzoite-related proteins. For example, deletion TgGRA83 disrupted bradyzoite differentiation and led to enhanced virulence [49]. The underlying mechanisms remained unclear, but they may relate to altered dissemination dynamics, as tachyzoites have a higher ability to spread widely throughout the host [50]. This dissemination included the ability of the parasites to breach biological barriers such as the placenta and the blood–brain barrier, leading to active invasion of fetal tissues and the central nervous system in adult mice. Although the *Nc*Δ*BPK1* group showed higher fertility and larger litter size compared with the WT, these differences are not attributable to NcBPK1 deletion, since mating and implantation occur before infection and variability in these parameters is common in this murine model due to factors such as incomplete synchronization of the Whitten effect, variability among males, and social competition between females.

Regarding the humoral immune response, a higher antibody production against *N*. *caninum* was observed in *Nc*Δ*BPK1*-infected mice, suggesting a widespread tachyzoite dissemination throughout the host. This may account for the fatal outcome of infection observed in this group. In addition, pregnant mice infected with *Nc*Δ*BPK1* tachyzoites exhibited a lower IgG1/IgG2a ratio compared with those challenged with WT parasites, indicating a stronger Th1 polarization. This would be typically more effective at controlling intracellular pathogens and promoting macrophage activation and IFN-γ production [51, 52]. However, our findings contrast with the prevailing view that a Th1-mediated immune response, particularly through IFN-γ production, confers protection against *N*. *caninum* by promoting parasite clearance [52–54]. The enhanced dissemination and uncontrolled replication of parasites observed in the *Nc*Δ*BPK1*-infected mice suggested a breakdown in immune regulation, potentially impairing the ability of the host to control infection while maintaining tissue integrity. Moreover, the excessive production of Th1-associated cytokines, including IFN-γ, may contribute to immunopathological damage by disrupting the balance between Th1 and Th2 responses, ultimately exacerbating disease severity.

One possible explanation is that *Nc*Δ*BPK1* tachyzoites replicate more rapidly due to a reduced capacity to convert into the bradyzoite stage. This would overwhelm the host immune defenses before they can effectively control the infection. This hypothesis is supported by the tachyzoite to bradyzoite conversion assay, which showed a delayed and reduced/lower expression of *NcSag4* in the *Nc*Δ*BPK1* strain compared with the WT. The *NcSag4* gene is a bradyzoite-specific marker [32], and its expression mirrors that of its *T*. *gondii* orthologue. In addition, analysis of *NcBpk1* expression during stage conversion showed a gradual increase in transcript levels, peaking at day 7. This pattern is consistent with a potential involvement of NcBPK1 in the bradyzoite developmental program and in processes linked to parasite persistence. The ability to modulate gene expression during stage conversion is essential for parasite persistence, as it enables cyst formation and long-term survival in host tissues. This aligns with data from *T*. *gondii*, where TgBPK1 is essential for the maintenance and stability of tissue cysts [18]. In fact, co-immunoprecipitation studies revealed that TgBPK1 interacts with key cyst wall and parasitophorous vacuole membrane components such as GRA8, GRA9, and MAG1 [18]. By extrapolating these findings, it is plausible that NcBPK1 plays a structural or regulatory role in cyst formation in *N. caninum.* Identifying the protein interactions and signaling pathways associated with NcBPK1 will be crucial for elucidating its precise role in the biology of this parasite in future research. Although our data are consistent with a role for NcBPK1 in regulating stage conversion, other functions cannot be ruled out. NcBPK1 belongs to the ROPK-like family, and in *T*. *gondii* several ROPKs have been shown to manipulate host cell signalling and immune responses. It is therefore possible that NcBPK1 also contributes to host–parasite interactions or immune modulation, and that these mechanisms may influence the phenotypes observed in the KO strain. Further studies will be required to investigate this possibility.

Deletion of *NcBpk1* appears to tip the balance between acute and chronic stages, favoring continuous replication and exacerbating disease outcomes. Interestingly, previous studies have reported adverse outcomes when using bradyzoite-specific antigens as vaccine candidates. For instance, immunization with a inactivated whole tachyzoite-bradyzoite mixture exacerbated the clinical signs of neosporosis in both congenital and cerebral mouse models [55]. This work proposed that vaccinated mice displayed rapid parasite dissemination and uncontrolled tachyzoite replication, which led to immune dysregulation and, ultimately, worsened disease outcomes, similar to what was found in this study upon infection with *Nc*Δ*BPK1* tachyzoites. Further investigations into the number and distribution of tissue cysts, particularly in the brains of infected mice, could help clarify whether the loss of NcBPK1 affects chronic infection and cyst persistence in vivo. In this context, determining whether the increased parasite burden observed in the central nervous system reflects the presence of actively replicating tachyzoites or tissue cysts would provide additional insight into the dynamics of parasite stage conversion and persistence. However, this analysis was not performed in the present study due to the difficulty in detecting tissue cysts in BALB/c mice, where bradyzoite-containing cysts are either absent or rarely form in the central nervous system.

Knowledge of IFN-γ-mediated responses in *N*. *caninum* infections remains limited. This contrasts with what is known in *T*. *gondii*, which has been shown to develop multiple strategies to counteract this key host defense mechanism [56]. These strategies include the secretion of effector proteins from rhoptries and dense granules, which are capable of manipulating host cell signaling, and the conversion to a bradyzoite stage, which is more resistant to IFN-γ. Therefore, we tested if our *Nc*Δ*BPK1* mutant, with diminished conversion to a bradyzoite-like stage, was less resistant to IFN-γ-mediated growth inhibition. Similar approaches have already been described in *T*. *gondii* [57]. Both the parental and the KO strains exhibited a similar reduction in parasite burden upon IFN-γ treatment, indicating that their replication is equally susceptible to this cytokine. These findings are consistent with observations in previous *NcRop40* and *NcGra7-*KO mutants of *N*. *caninum* [58]. Our results indicate that NcBPK1 is not directly involved in IFN-γ resistance. Instead, its role in immune modulation appears to be more related to the intrinsic parasite replication capacities than its ability to evade immune clearance.

The elevated expression of NcBPK1 observed in highly virulent isolates such as Nc-Spain7 was the rationale for its selection as a candidate gene for functional characterisation. Our findings, however, indicate that NcBPK1 does not function as a classical virulence factor promoting pathogenesis. Instead, the data suggest a regulatory role in restraining tachyzoite proliferation and supporting persistence. Upregulation of NcBPK1 in virulent isolates may therefore represent an adaptive mechanism to temper excessive replication in response to host immune pressure, promoting a balance between acute infection and long-term survival. In the absence of NcBPK1, this regulation is lost, leading to sustained tachyzoite proliferation, higher parasite burdens, and more severe disease progression. This highlights an unexpected divergence between expression associations and biological function, with NcBPK1 acting as a brake on replication rather than a driver of virulence.

## Conclusions

This study provides new insights into the role of NcBPK1 in *N*. *caninum* biology. NcBPK1 was initially selected for functional characterisation owing to its elevated expression in highly virulent isolates, yet deletion of the gene produced the opposite of the expected phenotype: enhanced parasite replication, higher brain parasite burdens, and reduced survival times in the murine model. These findings indicate that NcBPK1 does not act as a classical virulence factor promoting pathogenesis, but rather as a regulator that restrains tachyzoite proliferation and contributes to the balance between acute infection and long-term persistence. The impaired tachyzoite-to-bradyzoite transition observed in the knockout further supports a role in parasite persistence, which is essential for survival and transmission. Although the molecular mechanisms remain to be elucidated, this unexpected functional divergence highlights the importance of NcBPK1 in modulating parasite development under host pressure. Future studies should focus on identifying NcBPK1-associated protein interactions and signalling pathways, as well as exploring potential roles in host–parasite interactions, to further clarify its role in parasite biology.

## Supplementary Information

Supplementary file 1.

Supplementary file 2.

## Data Availability

Data supporting the main conclusions of this study are included in the manuscript.
