# Supplementary material for: Loss of NcBPK1 impairs bradyzoite differentiation and enhances virulence in Neospora caninum
Source: Parasit Vectors. 2025 Oct 21;18:422. doi: 10.1186/s13071-025-07076-4 (PMC12542182; doi:10.1186/s13071-025-07076-4)
Supplement: Supplementary file 1 [file 13071_2025_7076_MOESM1_ESM.pdf]

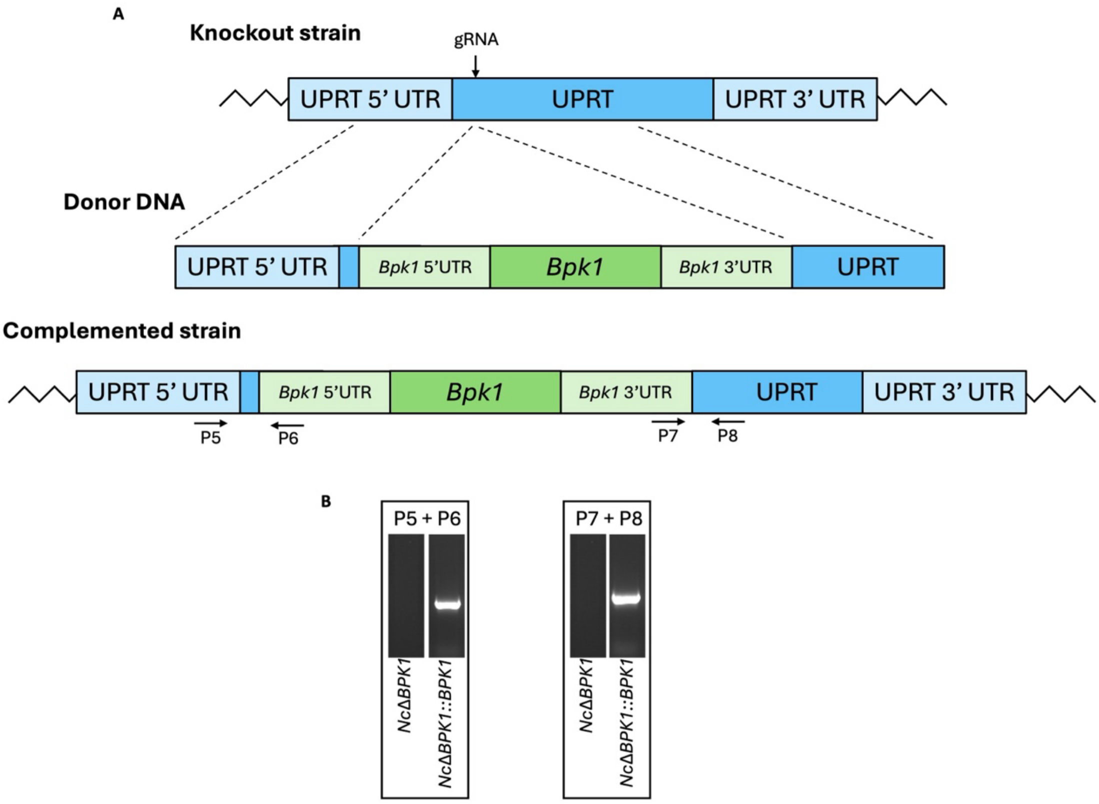

Supplementary Figure S1. Construction of the complemented strains by CRISPR/Cas9. A: Schematic diagram of NcBPK1 complementation of the knockout parasites by insertion into the UPRT locus. UPRT locus was disrupted by using one gRNA. Donor template has the exogenous copy of NcBpk1 with its corresponding UTR regions (Bpk1 5' UTR and Bpk1 3' UTR), flanked by fragments of DNA matching the regions around the cutting point by Cas9. Arrows indicate the position and orientation of primers used in diagnostic PCR. UTR, untranslated region. B: Successful integration into the *UPRT* locus was confirmed by PCR amplification using primer pairs P5 + P6 and P7 + P8. P5: TACGTTTCTCCCAAAATCCC. P6: TGGATTAGACCCTGAGACGC. P7: GACAAACATTCCGGATGAGG. P8: CTGCTTCATGAGCACCACAT.
