# Supplementary material for: Loss of NcBPK1 impairs bradyzoite differentiation and enhances virulence in Neospora caninum
Source: Parasit Vectors. 2025 Oct 21;18:422. doi: 10.1186/s13071-025-07076-4 (PMC12542182; doi:10.1186/s13071-025-07076-4)
Supplement: Supplementary file 2 [file 13071_2025_7076_MOESM2_ESM.pdf]

Supplementary Table S1. Primers used in the present work

Name	Sequence (5' – 3')	Description	Reference
BPK1 gRNA 1	TCTCTGGACGCACCTGAAAG	Disrupt BPK1 gene in 5' and 3' terminal ends	Present work
BPK1 gRNA 2	TGGTAGGGAACGGGAGTATG		
BPK1 Fw (P1)	AGAGTGCGCTCAAGGTGATT	Check correct deletion/complementation of NcBPK1	Present work
BPK1 Rv (P4)	AAGACCGCGATTTCAACAGA		
DHFR Fw (P3)	TCTGGCAGGCTACAGTGACA	Check correct deletion of the gene of interest	(19)
DHFR Rv (P2)	GCCTGGTATCTTTATAGTCC		
pSS013 Fw	CAAATGGCGACCTGCAGAGG	Check correct ligation of gRNA into pSS013 plasmid	Present work
UPRT gRNA	GCAGGAGGAAAGCATTCTGC	Disrupt UPRT gene in 5' terminal end	(31)
UPRT Fw1	tctagaggatccccgggtacCCAGCTGGTACTCTAGAAC	Amplification of the upstream flanking area from the Cas9 cutting site. sequence binding to the pUC19 plasmid is shown in lowercase	Present work
UPRT Rv BPK1	cgtgtttagcgacgaaagccCTGCTCAGTTGTCGCGTATC	Amplification of flanking area 5' of the Cas9 cutting point. Region that anneals with NcBPK1 in lowercase	Present work
UPRT Fw2	TACCGCCAGGTAATCCTTC	Amplification of flanking area 3' of the Cas9 cutting point. Region that anneals with pUC19 in lowercase	Present work
UPRT Rv	agtgaattcgagctcggtacGGCAGACAGAATTGAAGAC		
BPK1 Fw2	GGCTTTCGTCGCTAAACAC	Generation of the exogenous copy of NcBPK1 with UTR regions. Region that anneals with UPRT in lowercase	Present work
BPK1 Rv2	agaaggattacctggcggtaGAGAAGCGACAAGATGCCG		
NcDhfr Fw	ATCTGGGACAAGAATGTGAC	qPCR	(21)
NcDhfr Rv	CGAAGTGTCTCCACTGGA		
NcDhfr probe	Cy5-CGAGAGGTCGGAGACATCGGC-BHQ	dhfr-ts TaqMan probe	(21)
Nc5 probe	NCQ-PFAM-TGGTAGCGGTGAGAGGTGGGATACGTG-BHQ	Nc5 TaqMan probe	(21)
28S Fw	TGCCATGGTAATCCTGCTCA	qPCR	(27)
28S Rv	CCTCAGCCAAGCACATACACC		
Nc5 Fw	CCCAGTGCGTCCAATCCTGTAAC	qPCR	(27)
Nc5 Rv	CTCGCCAGTCAACCTACGTCTTCT		
18S Fw	GATACAGAACCAACCCACCTTCC	RT-qPCR	(33)
18S Rv	AGACCGAAGTCAAACGCGATC		
NcSag4 Fw	GATTTCAAGAAGCCGCTGGA	RT-qPCR	(32)
NcSag4 Rv	TGAGAACTTGTGTGTCGCCTGTT		
NcSag1 Fw	CGGTGTCGCAATGTGCTCTT	RT-qPCR	(32)
NcSag1 Rv	ACGGTCGTCCCAGAACAAAC		
